# Spatial distribution of hospital admissions for asthma in the central area of Asturias, Northern Spain

**DOI:** 10.1186/s12889-023-15731-7

**Published:** 2023-04-28

**Authors:** Verónica González-Iglesias, Isabel Martínez-Pérez, Valentín Rodríguez Suárez, Ana Fernández-Somoano

**Affiliations:** 1 Departamento de Medicina, IUOPA-Área de Medicina Preventiva Y Salud Pública, Universidad de Oviedo. C/Julián Clavería S/N, 33006 Oviedo (Asturias), Spain; 2 Dirección General de Salud Pública, Consejería de Salud, Principado de Asturias, C/ Ciriaco Miguel Vigil, 9, 33006 Oviedo, Spain; 3 CIBER Epidemiología Y Salud Pública (CIBERESP), Instituto de Salud Carlos III, Avenida Monforte de Lemos, 3-5, 28029 Madrid, Spain; 4 Instituto de Investigación Sanitaria del Principado de Asturias (ISPA), Avenida Roma, S/N, 33001 Oviedo, Spain

**Keywords:** Asthma, Spatial distribution, Geographic Information System, Disease mapping

## Abstract

**Background:**

Asturias is one of the communities with the highest rates of hospital admission for asthma in Spain. The environmental pollution or people lifestyle are some of the factors that contribute to the appearance or aggravation of this illness. The aim of this study was to show the spatial distribution of asthma admissions risks in the central municipalities of Asturias and to analyze the observed spatial patterns.

**Methods:**

Urgent hospital admissions for asthma and status asthmaticus occurred between 2016 to 2018 on the public hospitals of the central area of Asturias were used. Population data were assigned in 5 age groups. Standardised admission ratio (SAR), smoothed relative risk (SRR) and posterior risk probability (PP) were calculated for each census tract (CT). A spatial trend analysis was run, a spatial autocorrelation index (Morans I) was calculated and a cluster and outlier analysis (Anselin Local Morans I) was finally performed in order to analyze spatial clusters.

**Results:**

The total number of hospital urgent asthma admissions during the study period was 2324, 1475 (63.46%) men and 849 (36.56%) women. The municipalities with the highest values of SRR and PP were located on the northwest area: Avilés, Gozón, Carreño, Corvera de Asturias, Castrillón and Illas. A high risk cluster was found for the municipalities of Avilés, Gozón y Corvera de Asturias.

**Conclusions:**

The spatial analysis showed high risk of hospitalization for asthma on the municipalities of the northwest area of the study, which highlight the existence of spatial inequalities on the distribution of urgent hospital admissions.

##  Background

Asthma is a chronic respiratory disease characterised by inflammation of the airways. The most common symptoms are breathing difficulties or shortness of breath, wheezing, chest tightness, and coughing, leading to expiratory airflow limitation [1].

The Global Burden of Disease (GBD) had estimated that, in 2017, more than 272 million individuals in the world would suffer from asthma [2]. Another study, which grouped the pathology into three groups―physician’s diagnosis, clinical asthma, and wheezing when breathing―and took into consideration the answers to a questionnaire applied by the World Health Organisation (WHO), had estimated that the global prevalence of asthma would be 4.3, 4.5, and 8.6%, respectively, which suggests that there were about 623 million individuals in the world affected by asthma at some of its levels [3].

The incidence of asthma varies greatly between different countries. However, the greatest figures for this disease are found in developed countries, with Australia (21.5%), Switzerland (20.2%), the United Kingdom (18.2%), and the Netherlands (15.3%) being the first four countries in the list of most affected countries [3]. A study conducted in several European cities in 2018 estimated that between 5.5 and 72.4% of patients diagnosed with asthma were undergoing treatment [4]. In Spain, the prevalence of asthma was estimated between 5 and 14% [5]. A study conducted in the population of the Community of Madrid, Spain, estimated an increase of 14% in the incidence of asthma [6]. Another study conducted in Murcia, Spain, indicated that 56 and 44% of visits to paediatric emergency units corresponded to asthmatic attacks in boys and girls, respectively [7]. Asturias is one of the regions of Spain with the highest rates of asthma. Specifically, in 2015, the sanitary area of Avilés exhibited a rate of 11.44 points, standardised by age and sex per 10,000 inhabitants, ranking in the last quintile, with an excess of 115 hospitalisations potentially avoidable [8].

Although asthma can develop at any age, its onset usually occurs during childhood, with greater incidence in boys than in girls. This fact is reversed when reaching adolescence, in which women begin to exhibit greater incidence of this disease [9]. There are multiple factors, both external (environmental allergens, air pollutants, etc.) and internal (genetic factors, individuals’ lifestyles), that can cause asthma onset or exacerbate its symptoms. Although there is great evidence that environmental factors exert a great influence on this disease, much is still unknown about how the interaction between these factors and genetics affects the development of asthma [10].

Tobacco smoke is one of the factors that can increase the risk of developing asthma, and also the risk of suffering asthma attacks and exacerbations of the disease [11]. Exposure to tobacco smoke during or after pregnancy is also a risk factor for the development of asthma in children, increasing the risk of wheezing by up to 70%, and the number of asthmatic episodes by up to 85% [12]. Another determinant factor is exposure to environmental allergens such as pollen, which is a trigger for asthmatic episodes, especially in children [13]. Overweight and obesity are also associated with increased incidence of asthma, reaching an increase of up to 50% in individuals who are overweight or obese [14]. The work environment also influences the onset of this disease. Work-related asthma refers to asthma caused by exposure to different substances in the workplace. Two subtypes of work-related asthma can be distinguished, namely: occupational asthma, when the disease develops without previous symptoms; and work-exacerbated asthma, which occurs when there was a previous diagnosis of the disease, but its condition worsens [15].

Asthma exacerbation also depends on atmospheric conditions such as temperature, relative air humidity or concentration of atmospheric aerosols. A study conducted in Poland showed a significant correlation between the number of asthma exacerbation and the Universal Thermal Climate Index (UTCI), observed more frequent cases in patients aged over 65 years when air humidity increases [16]. A systematic review aiming the effect of meteorological variables on asthma hospital admissions an emergency department visits suggests that temperature and relative humidity but also thunderstorms were associated with hospital hospitalizations in adults [17]. A study in Taiwan concluded than a 1 °C temperature increase was a protective factor for asthma emergency visits both for paediatric and adult patients and refers the need of taking into account meteorological factors and air pollution on asthma acute exacerbation studies [18].

Finally, there are multiple studies in which exposure to air pollutants has been related to the risk of suffering respiratory diseases, including asthma [19–22]. Even maternal exposure to these pollutants can have short and long-term effects on the respiratory system of children [23, 24]. A meta-analysis that included 87 studies and assessed the relationship between air pollution and the incidence of asthma, indicated a remarkable correlation between two factors, namely: (a) ozone (O_3_), carbon monoxide (CO), nitrogen dioxide (O), sulphur dioxide (SO_2_), particulate matter ≤ 10 µm (PM_10_), and ≤ 2.5 µm (PM_2.5_); and (b) visits to emergency units and hospital admissions due to asthmatic crises [25]. It has been estimated that, globally, between five and ten million visits to hospital emergency units due to asthmatic crises can be attributed to PM_2.5_. This number represents between 4 and 9% of annual visits [26]. In addition, exposure to air pollutants not only increases the risk of visits to emergency units due to asthmatic crises, but also contributes to the development of chronic obstructive pulmonary disease (COPD) in patients who have previously suffered from asthma, which is known as COPD-asthma [27].

The application of geographic information systems (GIS) in public health studies has been increasing in recent years. With the use of GIS tools, it is possible to assess spatial distribution, the existence of clusters, spatial–temporal evolution of different diseases, and generate risk maps both for the consultation of the citizens and for creating plans to improve health services in risk areas. Thus, a study conducted in Jeddah, Saudi Arabia, assessed the distribution and existence of clusters for three diseases, namely: diabetes; hypertension; and asthma, and made it possible to implement health plans in this city in order to improve healthcare services in the locations that exhibited greater risk of these diseases [4]. Another study conducted in Germany assessed the spatial and temporal distribution of asthma in this country from 2009 to 2016. It allowed the implementation of specific control and prevention programmes in regions with greater incidence of asthma [28]. A study conducted in the metropolitan area of New York, USA, used GIS to map the distribution of asthma and assess its prevalence in children aged 13 to 17 years. The results obtained indicated greater prevalence of asthma in the poorest areas of the city [29]. Another study conducted in the city of Tehran, Iran, used GIS tools and machine learning techniques to detect areas prone to greater incidence of asthma due to environmental and spatial factors, and allow the development of preventive plans in these areas of high risk [30].

The present study used GIS tools to determine the distribution of asthma in the municipalities of the central area of Asturias, northern Spain, which is an area with a high population density and with important industrial sections. It was also intended to detect areas with greater incidence of this disease and generate possible hypotheses that could explain the distributions observed.

## Methods

This is an ecological study conducted at the census tract (CT) level using two data sources, namely: hospital records and population data. Hospital records were obtained from the Minimum Basic Data Set (MBDS) database which is regulated by current legislation [31], and corresponded to unscheduled (urgent) hospital admissions for J-45 (asthma) and J-46 (status asthmaticus), according to the International Classification of Diseases (ICD-10). The Health Service of the Asturias Government, responsible of the management of the MBDS database, approved the use of the database according to the Collaboration Agreement SV-PA-19–03. All records were hospital admissions (hospital stays), the MBDS database does not include emergency care that does not require hospitalization. The records corresponding to the 2016–2018 period were selected and grouped into a single disease, i.e., asthma. The data used corresponded to the four hospitals located in the centre of Asturias, namely: San Agustín University Hospital, in Avilés; Cabueñes University Hospital; Jove Hospital, in Gijón; and Central University Hospital of Asturias, in Oviedo, which were the reference hospitals for the population included in this study.

**Fig. 1:**
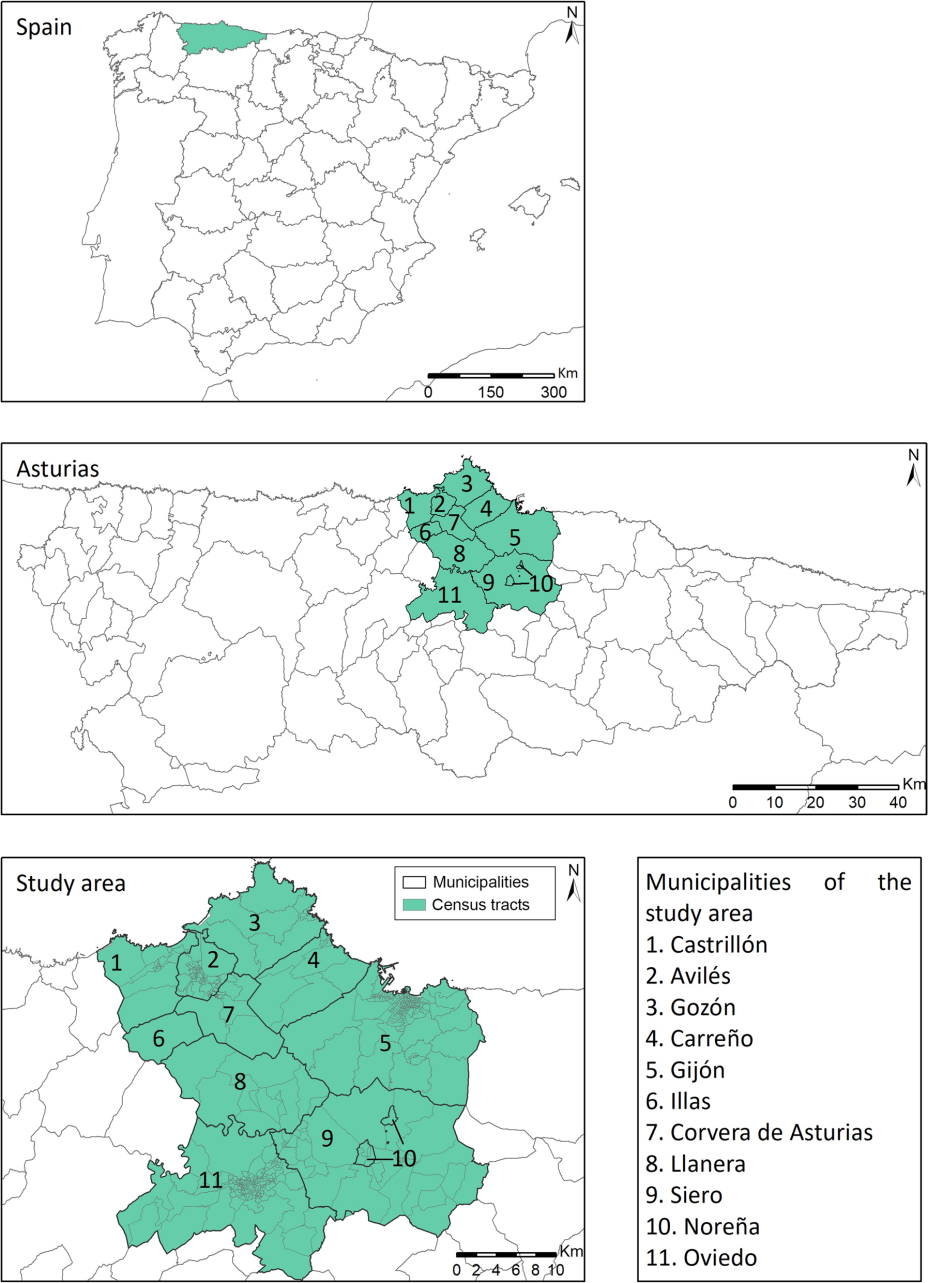
Location of the study area

The population data were obtained from the 2016 Municipal Register of Inhabitants, and grouped by age groups (less than 15; 15 to 39; 40 to 64; 65 to 84; and 85 or over) and sex. These data were provided by the Asturian Society for Economic and Industrial Studies (SADEI), with a total population of 705,968 inhabitants. The municipalities included in the present study were Avilés (80,114 inhabitants), Carreño (10,636 inhabitants), Castrillón (22,626 inhabitants), Corvera de Asturias (15,968 inhabitants), Gijón (273,422 inhabitants), Gozón (10,534 inhabitants), Illas (1,026 inhabitants), Llanera (13,846 inhabitants), Noreña (5,260 inhabitants), Oviedo (220,567 inhabitants), and Siero (51,969 inhabitants), which account for 67% of the total population of Asturias and 9.37% of its territory (Fig. 1).

The basic geographic unit used for the present study was the CT that corresponded to the smallest administrative unit that had information. The cartography used was that of 2016 provided by the Spanish National Institute of Statistics. The cartographic files used for the geocoding process were those provided by the municipalities of Oviedo, Gijón, and Avilés of specific doorway which cover both urban and rural areas; the data from the CartoCIUDAD project of the National Geographic Institute was used for the other municipalities. It has a good doorway coverage on urban areas but limited on rural areas. These datasets contain street number, street name, street type and ZIP code. It was also used the National Topographic Database BTN100, obtained from the National Geographic Institute to cover rural areas. The ArcGIS software was used for geocoding addresses in order to locate the residence addresses corresponding to each hospital admission. We create an Address locator as Single House type and a Multiple address locator based on villages and small towns for rural areas that had poor single house coverage. The geocoded records were linked to the CT by a spatial join and grouped by sex and age group.

The smoothed relative risk (SRR), calculated from the standardised admission ratio (SAR) by the indirect method, was used to assess the spatial distribution of admissions for asthma in the CT of the study area. SARs allow us to compare the total number of admissions observed for each CT with those we can expect according to their populations, and taking into account their age structure. However, they show specific problems when used in low populated areas (small area estimation problems). In that situation it is necessary to resort to more elaborate statistical estimators, as SRR, which considers the dependency between different units of analysis and facilitate to recover reliable risk estimates. The Poisson spatial model with random effects developed by Besag, York and Mollie [32] was used as a smoothing model, which took into account the spatial adjacency of other CT in the area. Simplified Laplace approximation was used for performing Bayesian inference, following the integrated nested Laplace approximation (INLA) procedure [33, 34]. The SRR values are expressed in points by 100. In addition, posterior risk probability (PP), or posterior probability that the SRR was greater than 100, was calculated. PP values above 0.8 indicated statistically significant excess of hospitalisations. These calculations were made using the Stata v14 and R version 3.6.1 programmes, and the INLA library. A spatial trend analysis was performed using the ArcGIS 10.4 geostatistical analysis tool. The trend analysis was performed in order to identify if there was any pattern on a specific direction of the map, this analysis provides a three-dimensional perspective of the data. It was performed as a second-order polynomials and plotted on south-north/ west–east directions. Pearson’s correlation coefficient measures the strength of a linear association between two variables and was added to the plots as ‘*r*’ value.

Finally, the Moran’s Index and the local indicator of spatial association (LISA) were used for the analysis of spatial clusters. Moran’s Index measures the spatial autocorrelation between the smoothed relative risks throughout the study area, and contrast the hypothesis of absence of global spatial autocorrelation versus the existence of spatial autocorrelation. The calculation of LISA let us detect if there is a possible spatial autocorrelation in a certain subset of spatial units. If it is statistically significant and positive, it confirms the presence of a cluster of similar values around the spatial unit, but, if it is negative, there will be a cluster of different values around the corresponding spatial unit (spatial outliers). Local Moran’s I analysis was performed under “Contiguity edge corners” as spatial relationship matrix, which is the most appropriate focus and provided the most understandable results for the study area. This approach means that polygons that share an edge or a corner will be included in computations for the target polygon. If any portion of two polygons overlap, they are considered neighbours and will be included in each other's computations. The tools used to that end were ArcGIS Spatial Autocorrelation (Moran’s I) and Cluster and Outlier Analysis (Anselin local Moran’s I).

## Results

There were 2,330 unscheduled hospital admissions for asthma during the study period and we were able to geocode 2,324 cases (99.7%), of which 1,475 (63.46%) corresponded to women and 849 (36.56%) to men. The age groups with the highest percentage of cases were <  = 14 years old, and 65 to 85 years old, with 32.10 and 32.83% of admissions, respectively (Table 1). In women, the age group with the highest percentage of cases (39.46%) was 65 to 85 years old. On the other hand, for men, it was <  = 14 years old, with 54.30% of cases (Table 1). The number of admissions by age group was always higher in women, excepting those aged less than 14 years, for whom this situation was reversed, given that men had higher number of admissions (Table 1).

**Table 1:** Distribution by age of the number of unscheduled admissions for asthma

	**TOTAL**		**WOMEN**		**MEN**	
Age group	Nº of cases	%	Nº of cases	%	Nº of cases	%
< = 14	746	32.10	285	19.32	461	54.30
[15—40)	177	7.62	112	7.59	65	7.66
[40—65)	350	15.06	243	16.47	107	12.60
[65—85)	756	32.53	582	39.46	174	20.49
> = 85	295	12.69	253	17.15	42	4.95
Total	2324	100	1475	100	849	100

**Fig. 2:**
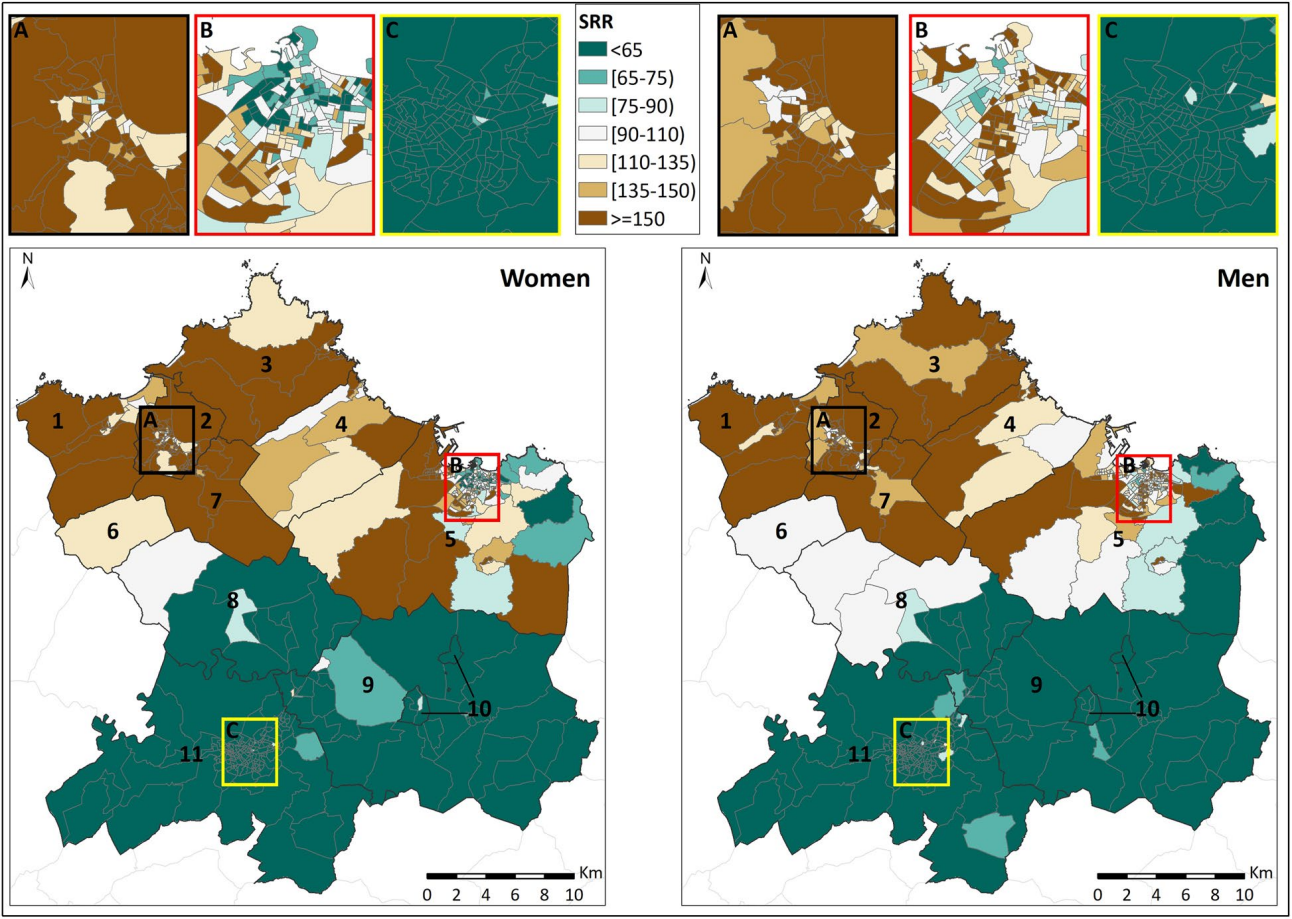
Smoothed relative risk (SRR). 1: Castrillón; 2: Avilés; 3: Gozón; 4: Carreño; 5: Gijón; 6: Illas; 7: Corvera de Asturias; 8: Llanera; 9: Siero; 10: Noreña; and 11: Oviedo

There was a clear difference in the spatial distribution of admissions for asthma between the north and south of the study area for both men and women. The southern municipalities (Oviedo [11], Siero [9], Noreña [10] and Llanera [8]) exhibited low SRR values, i.e., below 65 in the majority of the CT. On the other hand, the municipalities of the northern zone (Avilés [2], Gozón [3], Carreño [4], Corvera de Asturias [7], Castrillón [1], Illas [6] and the western part of Gijón [5]) exhibited high SRR values, exceeding 150 in most CT of these municipalities (Fig. 2).

The assessment of the smallest CT corresponding to the three largest urban areas of the region (Gijón [B], Oviedo [C] and Avilés [A]) indicated a clear pattern of high values in Avilés (above 150 in the great majority of CT for both sexes) and low values in Oviedo (below 65 in the vast majority of CT, also for both sexes). In the case of the urban area of Gijón, as in the municipality as a whole, great heterogeneity was observed in the distribution of the SRR values in the CT. There seemed to be a predominance of CT with high values (above 110) only in the case of men.

**Fig. 3:**
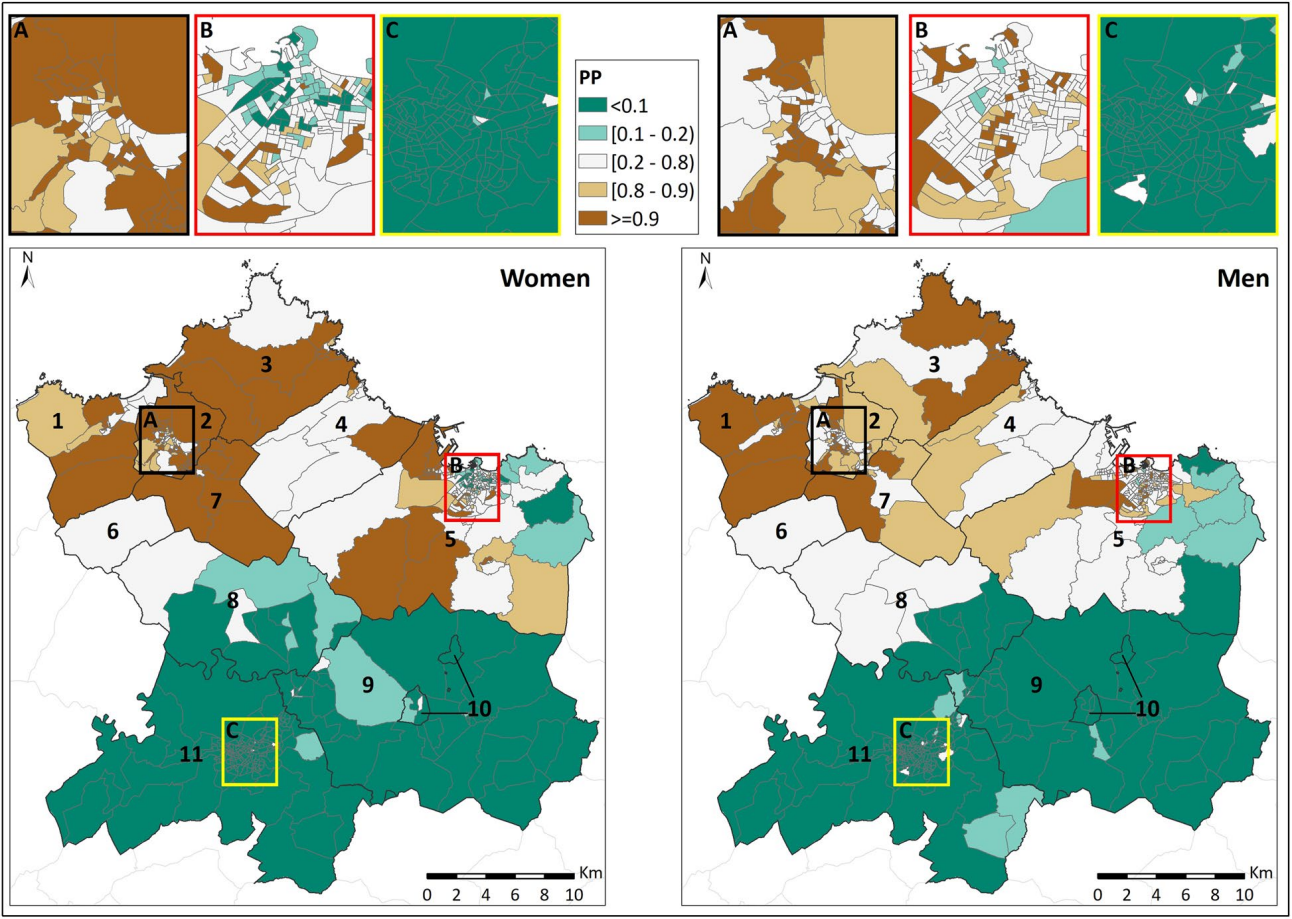
Posterior risk probability (PP). 1: Castrillón; 2: Avilés; 3: Gozón; 4: Carreño; 5: Gijón; 6: Illas; 7: Corvera de Asturias; 8: Llanera; 9: Siero; 10: Noreña; and 11: Oviedo

The PP followed a similar distribution of the SRR, with low values (below 0.2) in the municipalities of the southern zone (Oviedo [11], Siero [9], Noreña [10], and part of Llanera [8]), and high values (always above 0.8) in the northern one (Avilés [2], Gozón [3], Carreño [4], Corvera de Asturias [7], Castrillón [1], and Illas [6]) for both sexes (Fig. 3).

**Fig. 4:**
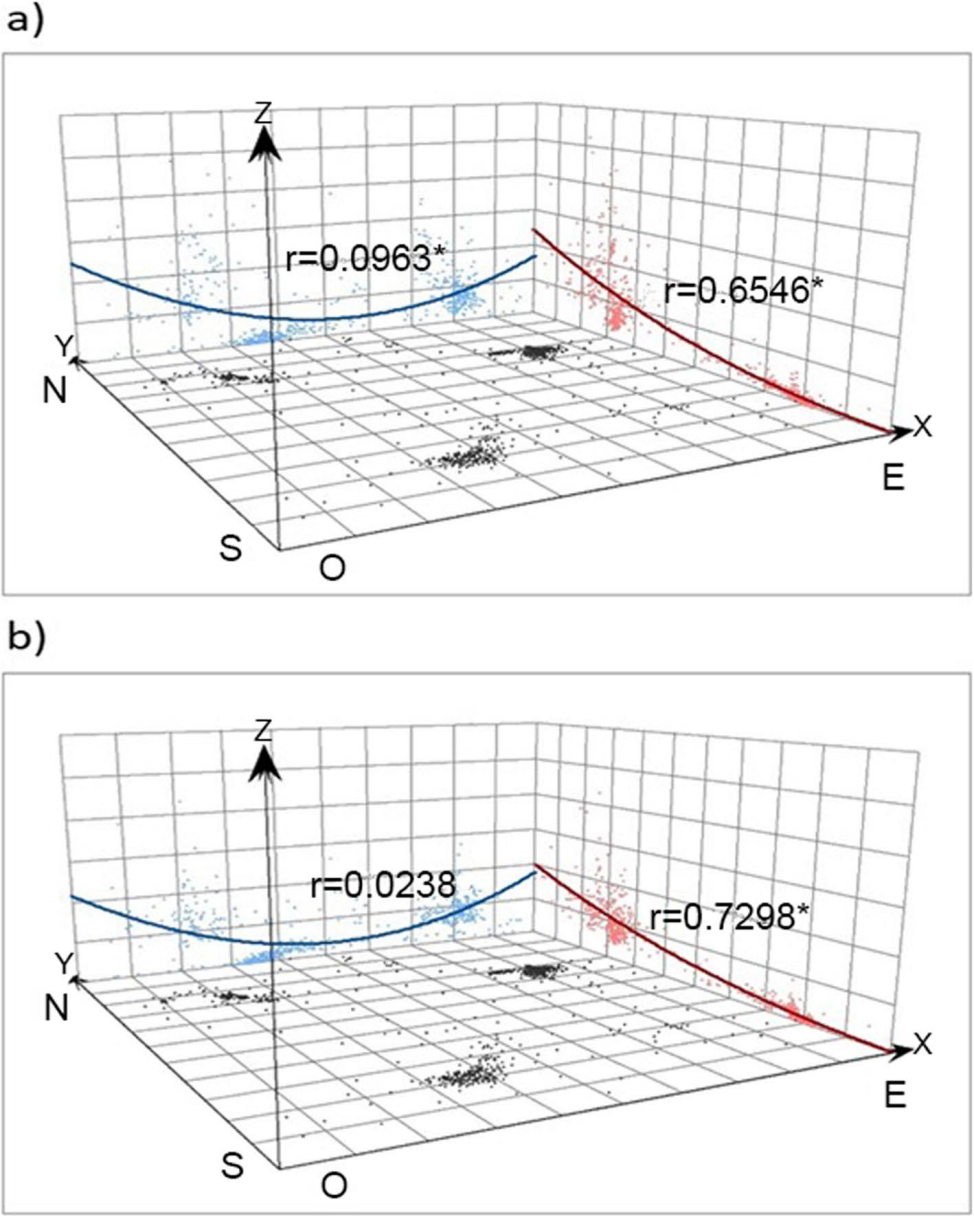
Spatial trend. **A**) Women; **b**) Men; r: Pearson's correlation coefficient; * statistically significant

The values of the CT in urban areas (Gijón [B], Oviedo [C], and Avilés [A]) exhibited the same pattern observed in the municipalities, with low values in Oviedo, for both men and women (below 0.1 in the vast majority of the CT), and high values in Avilés, where there was certain difference between women and men, since the PP values in the CT of women were generally higher than in the case of men. In the case of the urban area of Gijón, great heterogeneity was observed in the PP values, especially in the case of women. The spatial trend graphs for SRR illustrate statistically significant arrangement of higher values, both for women and in men, as we move towards the north of the studied area (Fig. 4).

At a global level, a positive, moderate and statistically significant spatial autocorrelation was observed in the study area (Table 2). This result indicates the existence of similar risks of hospital admission for asthma in neighbouring geographic units, in other words, spatial clustering.

**Table 2:** Summary of global Moran’s I

Moran’s Index		Expected Index		Variance		z-score		*p*-value	
W	M	W	M	W	M	W	M	W	M
0.6899	0.6104	-0.0018	-0.0018	0.0006	0.0006	28.2912	25.1469	< 0.0001	< 0.0001

*W* Women, *M* Men

**Fig. 5:**
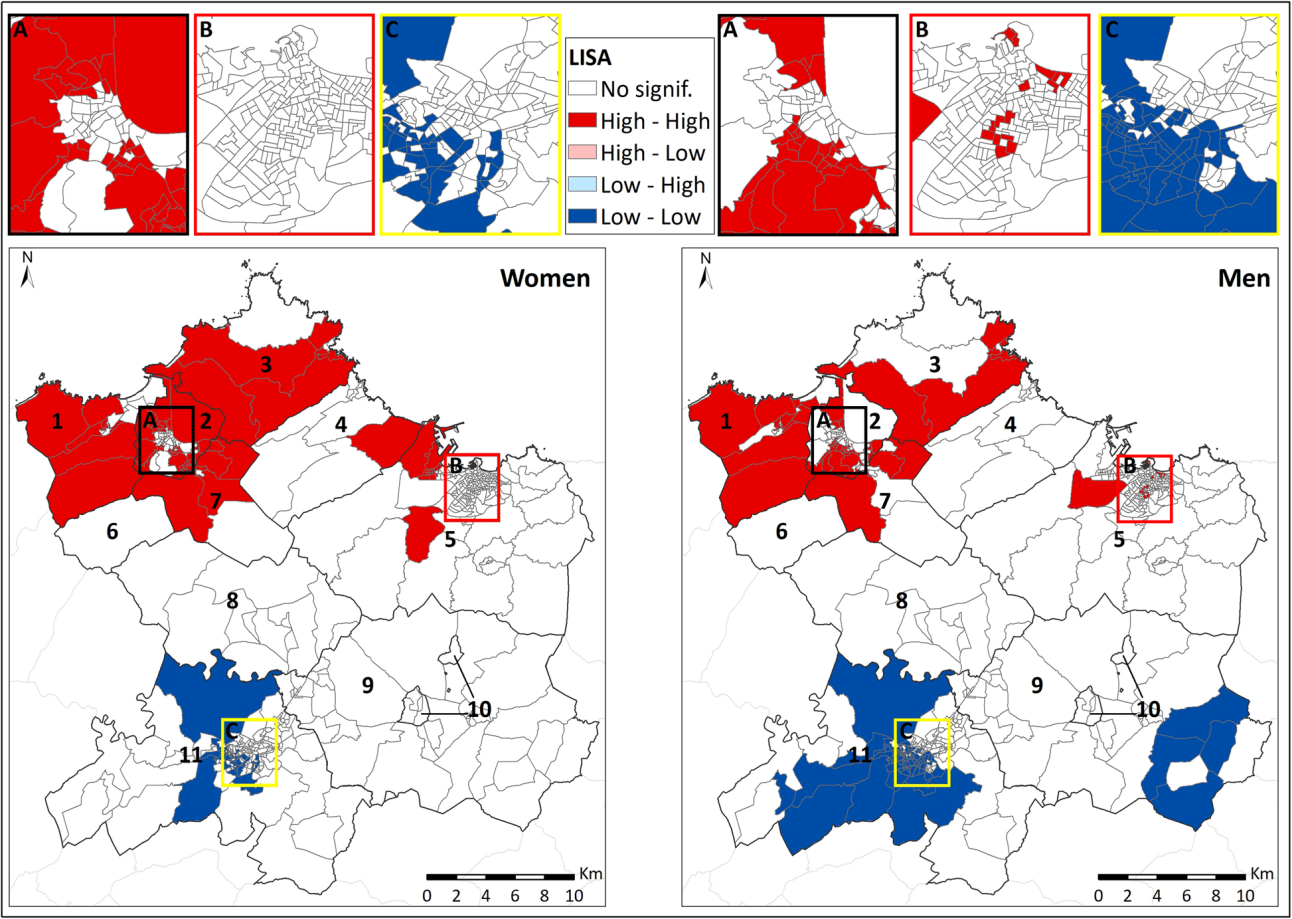
Spatial clusters—Anselin local Moran’s I (LISA). 1: Castrillón; 2: Aviles; 3: Gozón; 4: Carreño; 5: Gijón; 6: Illas; 7: Corvera de Asturias; 8: Llanera; 9: Siero; 10: Noreña; and 11: Oviedo

The distributions of high SRR values in the northern zone, and low in the southern zone were associated with the cluster of CT that exhibited high and low values, respectively, both being statistically significant (Fig. 5).

The cluster of CT with high SRR values occurred mainly in the municipalities located in the north of the studied area (Avilés [1], Gozón [3], and Corvera de Asturias [7]). In the case of women, there was also a cluster of high values in the western area of Gijón (5) and in some CT of the Municipality of Carreño (4). On the other hand, for men, although the CT of the western area of Gijón (5) exhibited high SRR values, they hardly exhibited statistically significant clusters. The low SRR values (< 65) observed in the Municipality of Oviedo (11) indicated a statistically significant spatial clustering, which was more evident in the case of men. There was also an area in the east of the Municipality of Siero (9) that exhibited a cluster of CT with low SRR values, and was statistically significant (< 65) only in the case of men.

Regarding the urban areas of the municipalities, there was a cluster with statistically significant high values (above 150 in most of the CT) in the urban nucleus of Avilés (A), more evident in women, excluding the CT located near the city centre that did not exhibit statistically significant clustering. The urban centre of Oviedo (C) had a statistically significant cluster of CT with low SRR values (< 65) for men. In the case of women, this clustering was not so wide. In Gijón (B), as mentioned above, there was great heterogeneity in the SRR values in the case of women; therefore, there were no clusters. On the other hand, for men, although there was also a significant heterogeneity, the SRR values were generally higher, and the cluster analysis indicated some CT with statistically significant high values (> = 150).

## Discussion

The cumulative incidence of unscheduled hospital admissions for asthma from 2016 to 2018 was 0.33% for the total population of the central area, this incidence being higher in women (0.4%) than in men (0.25%). The spatial distribution of these admissions in the central area of Asturias indicated a clear north–south pattern with the highest values and, therefore, the highest risk in the northern municipalities (Avilés, Gozón, Carreño, Corvera de Asturias, Castrillón, and Illas). This result was corroborated by the subsequent cluster analysis, which revealed the existence of CT clusters with high risk values, not due to chance, in the municipalities of Avilés, Gozón, and Corvera de Asturias.

The results obtained assessing the number of cases by age groups and sex are consistent with those of other studies that found higher prevalence of this disease in women [9]. This situation was reversed in adolescents (< = 14 years), since men had higher number of asthma cases. Some authors have pointed to a regulatory role of hormones to explain this change [34].

A possible explanation for the heterogeneous geographical distribution of the incidence of asthma in this study could be air pollution, since the areas that seemed to be at greater risk of hospital admissions for asthma where those with many industries in Asturias [35]. This fact entails greater amounts of polluting substances being emitted, both from the industry and from the road traffic associated with it [36]. Thus, living close to the industries that are present in the study area, mainly metal production and processing installations, have been associated with lung cancer risk in the literature [37]. Air pollutants exert a great influence as asthma triggers, both for promoting the onset of the disease and exacerbating its symptoms. A study conducted in Atlanta, USA, assessed the association between asthma morbidity and the combination of different types of pollutants, and found greater asthma morbidity on days when there was high or moderate concentration of primary pollutants coming from vehicles, and secondary pollutants [38]. Another study conducted with data on admissions to emergency units for respiratory diseases, including asthma, in five European cities, found an important association between high concentration of ultrafine particles and admissions to the emergency units, especially of young individuals aged 0 to 14 years [39]. A meta-analysis that assessed the association between the concentration of NO_2_ and various respiratory diseases in China indicated that the increase in 10 µg of NO_2_ corresponded to a 1% increase in hospital admissions [40]. Improving the epidemiological surveillance system for air quality in the region could help to reduce the intraregional heterogeneity detected.

The geographical disparity found on hospital admissions for asthma on this study may be influence also by meteorological aspects, as shown on several studies that take into account climate variables and their influence on asthma exacerbations [16, 17, 41]. The CTs on our study that present a higher incidence are coastal areas with high relative humidity and warm temperatures that could be influencing admissions due to asthma.

The existence of unequal distributions of hospital admissions due to asthma has also been observed in other studies. In Germany, urban areas had higher incidence of asthma than rural areas [28]. Likewise, in a study conducted in Tehran, Iran, a strong spatial correlation was observed between the distance to parks and streets and the prevalence of asthma [30]. Similarly, a study conducted to assess the spatial distribution of three diseases in Jeddah, Saudi Arabia, found clusters with high values for asthma in urban areas, and clusters with lower values in less urbanised peripheral areas [4]. The central area of Asturias concentrates almost 70% of the population of the region in a quite small territory, and the higher incidence was observed on suburban areas and industrial land. In addition, the distribution of the risk of admission due to asthma observed in the present study is in line with the distribution of other diseases associated with environmental pollution, such as acute myocardial infarction, angina pectoris, and chronic obstructive pulmonary disease, published in other studies [42, 43].

Socioeconomic factors could be another explanation for the distribution of this disease [44]. Accordingly, the areas with the highest deprivation in the three main cities of the study area, published in the web app http://www.uv.es/medea/medeapp.html (accessed on 20 Dec 2022) largely coincide with areas with high incidence rates. Being a smoker and being obese or overweight are other risk factors for asthma, thus increasing the incidence of this disease [11, 14]. Asturias is a region where the obesity rate is one of the highest in Spain and the percentage of heavy smokers is also one of the highest in the country, which contributes to the burden of disease [45].

One of the main limitations of the present study was that, due to the lack of studies addressing the association with pollutants and the diversity of factors that affect the onset of the disease, only the distribution of hospital admissions due to asthma in the central area of Asturias could be assessed. However, this distribution could not be attributed to a specific cause but to many different ones that act together. It should also be taken into account that the addresses considered in the study might not be the place where those individuals spent most of their time.

## Conclusions

A cluster of CT with high risk of hospital admissions due to asthma was observed in the north-western part of the study area. This finding highlights the existence of spatial inequalities in health possibly indicating different environmental exposures. It also draws attention to the need of conducting further in-depth studies in these areas, in order to know the cause of this high incidence of hospital admissions and perform actions to control these factors, thus improving the epidemiological condition in the municipalities assessed.

## Data Availability

The datasets generated and/or analysed during the current study are not publicly available due to privacy issues given the small size of the units of study, but are available from the corresponding author on reasonable request.

## Abbreviations

CO: Carbon monoxide
COPD: Chronic obstructive pulmonary disease
CT: Census Tracts
GBD: The Global Burden of Disease
GIS: Geographic information systems
ICD-10: International Classification of Diseases
INLA: Integrated nested Laplace approximation
LISA: Local indicator of spatial association
MBDS: Minimum Basic Data Set
NO_2_: Nitrogen dioxide
O_3_: Ozone
PM_10_: Particulate matter ≤ 10 µm
PM_2.5_: Particulate matter ≤ 2.5 µm
PP: Posterior risk probability
SADEI: Asturian Society for Economic and Industrial Studies
SO_2_: Sulphur dioxide
SRR: Smoothed relative risk
WHO: World Health Organisation
